# Effect of Surface Treatment on Physical and Tensile Properties of Borassus Fruit Fibers

**DOI:** 10.1155/ijbm/5583605

**Published:** 2025-02-06

**Authors:** K. Boimau, J. Bale, N. P. G. Suardana, I. P. Lokantara, D. G. H. Adoe, Y. Pell

**Affiliations:** ^1^Department of Mechanical Engineering, Faculty of Science and Engineering, Universitas Nusa Cendana, Kupang, Indonesia; ^2^Department of Mechanical Engineering, Faculty of Engineering, Udayana University, Denpasar, Bali, Indonesia

**Keywords:** Borassus fruit fibers, NaOH treatment, tensile properties, wettability

## Abstract

The physical and tensile analysis of NaOH-treated Borassus fibers is carried out to expand and open up further possibilities for their use as reinforcement in composites. This study will describe the NaOH treatment procedure on fibers utilizing the batch reactor approach, as the soaking method is typically used in this case. The physical analysis shows that the wettability behavior effect of NaOH was led by using 5%, 10%, and 15% of NaOH solutions. The result of each treatment using NaOH was then characterized by using SEM-EDX to show the morphology and mechanical properties of the material before and after alkali treatment. Otherwise, FTIR was used to identify the functional group before and after alkali treatment. The tensile properties of the Borassus fibers (raw and treated) were also discussed. The wettability test was carried out by using the contact angle measurement technique. All samples (natural and treated fibers) showed a contact angle of less than 90°, which is associated with the hydrophilic surface properties. This result indicated that this treated fiber has polar properties. The increase of NaOH percentage in the treated samples caused a decrease in the contact angle. This phenomenon was related to the SEM image of natural and treated fibers. SEM result shows the cleaning effect of nonpolar components from the fiber surface and the roughening of the surface during the NaOH treatment. The result of FTIR indicates that several functional group components, such as impurities, lignin, and hemicellulose, were leaching out from row fibers after alkali treatment. The mechanical test results indicate improved tensile properties due to the NaOH treatment. Moreover, the samples from the fiber treated with 10% NaOH indicated the highest tensile strength.

## 1. Introduction

In recent years, natural fibers have been used for a wide range of applications in composite technology due to their environmentally and economically friendly characteristics such as biodegradability, nontoxic properties, renewable resources, lower greenhouse gas and pollutant emissions, light in weight, and low cost [1–5]. Natural fiber composites' advantages include high specific stiffness and mechanical strength, reduced energy consumption, low density, and low hardness, which minimizes the wear of the processing equipment [6]. It is also known that the use of natural fibers as reinforcing materials for polymer composites can reduce negative carbon footprint. The use of natural fibers has advantages, such as reducing CO_2_ emissions [7].

However, the main drawback of natural fiber–reinforced polymer composites is the incompatibility between the hydrophilic natural fibers and the hydrophobic polymer matrix. This limitation leads to being undesirable for composite applications. Thus, it is necessary to modify the fiber (surface) properties by chemical treatment methods to reduce the hydrophilic nature of the fiber, improving adhesion between fiber and matrix [8–12]. The alkali treatment was found to be an inexpensive and effective method to remove most of the impurities existing on the fiber surface and part of the amorphous component (hemicellulose), thus resulting in better mechanical and thermal properties [13–15].

Borassus or Palmyra Palm (Borassus flabellifer L.) is economically advantageous and generally found in the tropical regions of Asia, especially in India and Indonesia. Borassus is a long-life tree that can live up to 100 years, reaching a height of 30 m, with a canopy of leaves and a large trunk resembling a coconut tree [16]. Borassus fiber is extracted from the waste of ripened Borassus fruits. Hence, it is inexpensive, abundantly available, eco-friendly, and essential for exploring the potential utility of green composites worldwide.

Various research works are being carried out with the Borassus fruit fibers; for example, research about the Borassus fruit fibers' structural characterization and tensile properties has been reported [17]. The results have shown that alkali treatment resulted in the solubilization of hemicellulose, leading to reduced content in the fibers. The mechanical tests revealed that tensile properties are higher for treated fibers than for raw fibers.

Boopathi, Sampath, and Mylsamy [18] evaluated the effect of alkali treatment on the Borassus fruit fibers. They concluded that 5% alkali-treated fibers produced better mechanical properties and could be used as reinforcement in composite applications. This result is related to our previous work, where alkali-treated Borassus fruit fibers have an impact on the tensile properties of polyester composites. The Borassus fiber has been reviewed and summarized to identify gaps in the earlier research work. It provides resourceful data for future work as reinforcement in the natural fiber–reinforced polymer composites [19, 20].

The concept of Borassus fibers–based polymer composites has been widely investigated. Sudhakara et al. [21] found that the mechanical properties of Borassus fruit fibers–PP composites are compared to jute–PP and sisal–PP and superior to the coir–PP composites. From the perspective of thermal properties, Ramanaiah, Prasad, and Reddy [22] revealed that the fiber volume of Borassus fruit fibers can significantly improve the thermal insulation capability of the composite. Borassus fruit fiber's good thermal insulating properties open up opportunities for energy-saving applications such as building facilities (air-conditioners), automobile interior parts, and electronic packages.

The challenges of Borassus fiber–reinforced polymer composites are the hydrophilic nature and poor thermal properties of Borassus fiber. Furthermore, Borassus fiber has a promising future due to its properties, which will be utilized for the following applications: automotive, machinery, construction, etc.

The recent investigation on the hydrophilic nature of Borassus fruit fibers as an essential parameter remains a challenge and has not been reported broadly. In the present work, we further investigated the effect of alkali treatment (for 5%, 10%, and 15%) on physical and tensile properties. This work aims to fill the gap by performing a wettability test, Fourier transform infrared (FTIR), and scanning electron microscopy (SEM) on Borassus fruit fibers as reinforcement of natural fiber–reinforced polymer composites.

## 2. Experimental Procedure

### 2.1. Materials

**Figure 1:**
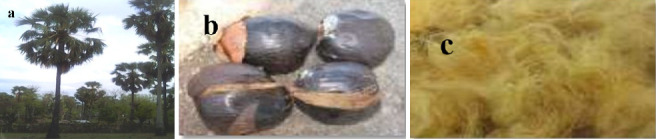
Photographs of (a) Borassus tree, (b) Borassus fruit, and (c) Borassus fruit fibers.

The Borassus flabellifer fibers were extracted from dried ripened fruits. Two types of fiber, long coarse and short fine fibers, were found. Both types of fibers were thoroughly washed with tap water, followed by distilled water, and dried in the sun for a week. Then, the fibers were dried in a hot air oven at 105°C for a period of 24 to remove the moisture. The dried fibers were treated with 5%, 10%, and 15% NaOH at room temperature for 2 hours. Then, the fibers were washed with distilled water repeatedly until they showed no residual NaOH. Finally, the fibers were washed with distilled water before drying in a hot air oven at 70°C for 24 h.  Figure 1 presents the photographs of the Borassus tree, Borassus fruit, and Borassus fruit fibers. The plan view of hole patterns is shown in Figure 1.

### 2.2. Physical Analysis

The physical analysis of the fibers was carried out on a wettability test to identify the contact angle and the droplet's shape. The optical technique to measure the contact angle was used to characterize fiber–liquid interactions. Single fibers (raw and treated) of 10 cm were dripped with 10 drops of a liquid matrix in three rows, and the fiber was placed on a jig (holder) made of wood. The droplets were seen in an optical microscope with a lens magnification of 40x, and the jig was positioned perpendicular to the microscope. The droplet test sample was photographed, and the angle was analyzed using the ImageJ program. The FTIR spectra of all the samples were run on Shimadzu IR Prestige-21. FTIR spectroscopy is used to investigate the composition of the samples. The samples were ground into powder and mixed with potassium bromide (KBr), and then the transmittance spectra were recorded in wavelengths between 500 and 4000 cm^−1^. The Scanning Electron Microscope (SEM) of the surface of the fibers were also recorded.

### 2.3. Tensile Properties

The tensile properties, such as stress, elasticity modulus, and % elongation at the break of the fibers, were determined using the ASTM D3379 test standard with a universal testing machine at a cross-head speed of 5 mm/min. In each case, five samples were used for each fiber condition (raw and treated), and the average was reported.

## 3. Results and Discussion

This experiment investigates Borassus fruit fibers' physical and tensile properties (raw and NaOH-treated). The percentage of NaOH treatment is set to 5, 10, and 15, respectively. The use of NaOH can remove impurities such as hemicellulose, lignin, waxes, and pectin [23].

### 3.1. Fiber Diameter

**Figure 2:**
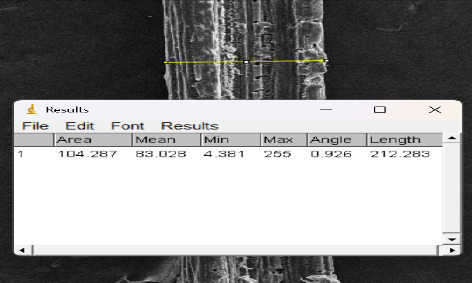
Measurement of Borassus fruit fibers.

Twenty-five samples of Borassus fruit fibers of each condition were measured (Figure 2) and the results are given in Table 1.

From the measurement, it was shown that the diameters of the fibers were decreased with respect to the increase in NaOH treatment percentage due to the fact that the NaOH treatment removed the impurities from the fiber surface. Furthermore, Table 1 shows that the fiber diameter decreases as the NaOH concentration increases, whereas fibers treated with 15% NaOH have a smaller fiber diameter than other treatments. In general, alkali treatment will result in the degradation of some of the cellulose in the fiber, resulting in a reduction in fiber diameter [23–28].

### 3.2. Physical Analysis

**Figure 3:**
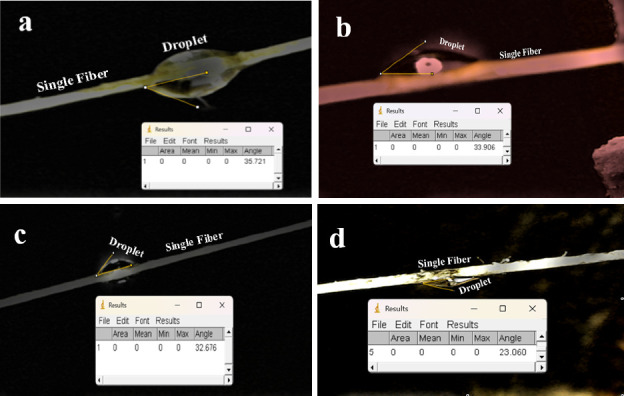
Photographs of contact angle: (a) raw fiber, (b) 5% NaOH-treated, (c) 10% NaOH-treated, and (d) 15% NaOH-treated.

The physical characterization of adhesion behavior can be predicted from the wettability of the fibers. The adhesion behavior's physical characteristics can be anticipated by examining the wettability of the fibers. In assessing the fiber's wetting behavior, an investigation was conducted on both the shape of the contact angle and the droplet, which were determined by using polyester liquid. Then, the image was processed by using the ImageJ software. The result is shown in Figure 3.

**Figure 4:**
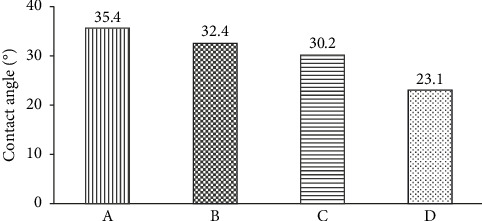
Contact angle (A) raw fiber, (B) 5% NaOH-treated, (C) 10% NaOH-treated, and (D) 15% NaOH-treated.

Figure 3 shows the oval-shaped droplets for all alkali treatments. This picture in Figure 3 also shows that the fibers without NaOH treatment (raw) have the highest contact angle compared to fibers with NaOH treatment. In contrast, the fibers with the lowest contact angle are treated with 15% NaOH. This low contact angle indicates that the fiber can be wetted well, which will impact a strong fiber–matrix interfacial bond. On the other hand, a high contact angle indicates that the fiber has poor wettability, which results in weak fiber–matrix interfacial bonds. All the values were measured (recorded) in a dark place, and five samples were tested for each composite. The contact angle of the composites is calculated and presented in Figure 4.

**Figure 5:**
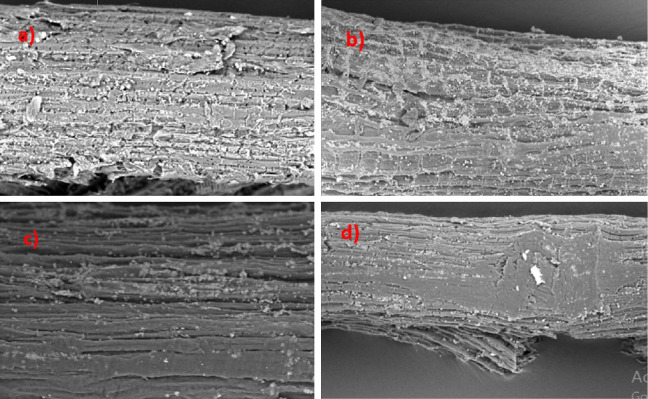
SEM image of (a) raw fiber, (b) 5% NaOH-treated, (c) 10% NaOH-treated, and (d) 15% NaOH-treated. All SEM image was conducted using 700x magnification.

Generally, the highest contact angle is recorded on smooth surfaces, and the lowest is on rough surfaces. This result indicates that the raw fiber was hydrophobic with low polarity, so it was challenging to get it. The existence of a nonpolar material, such as lignin, on the fiber surface caused the raw fiber to become hydrophilic. During the NaOH treatment, the nonpolar components were cleaned from the fiber surface, as observed in the SEM and presented in Figure 5.

**Figure 6:**
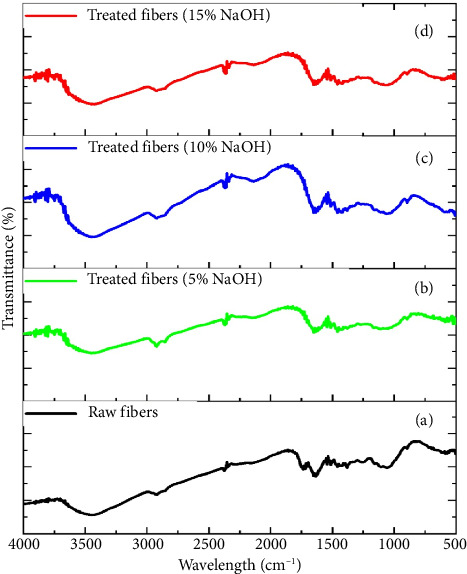
FTIR spectra: (a) raw fiber, (b) 5% NaOH-treated, (c) 10% NaOH-treated, and (d) 15% NaOH-treated.

Based on the SEM image in Figure 5(a)), it can be seen that the surface of the raw fiber still looks rough and uneven. In the 5% NaOH treatment, it was only able to remove some of the impurities, while in the treatment using 10% NaOH, the fiber surface looked cleaner, more regular, and smoother. In the 15% NaOH treatment, it can be seen that the fiber structure becomes damaged and brittle, as indicated by the signs of degradation by NaOH. It is known that the higher the concentration of NaOH used (above 10%), the more damage it will cause to the fiber structure [29, 30]. This phenomenon agreed with the results reported by early workers [16–19]. FTIR techniques were used to confirm the changes in the composition of NaOH treatment of the Borassus fruit fibers.  Figure 6 shows the FTIR spectra of the raw and NaOH-treated fibers.

Based on Figure 6, it can be seen that raw fiber has a distinctive peak, where the peak at 3330 cm^−1^ is the peak of OH stretching, which is a hydroxyl group related to the presence of cellulose, hemicellulose, and lignin in raw fibers. The peak at 2920 cm^−1^ is the peak of CH stretching, which is a methyl and methylene group from the lignocellulose structure. The peak at 1735 cm^−1^ is the C=O stretching group, indicating the presence of esters or carbonyls from hemicellulose. The peak at 1600 cm^−1^ is the C=C aromatic group, indicating the presence of an aromatic lignin structure. The peak between 1050 cm^−1^–1250 cm^−1^ is the C-O stretching group, indicating the presence of alcohol and ether content from the components in the polysaccharide structure. FTIR spectra for each alkali treatment of 5%, 10%, and 15% each produced three typical peaks, namely, the peaks with slightly different percentage intensities. The three peaks were observed at (1) the peak intensity of 3330 cm^−^1 (OH group), where the alkali treatment is thought to cause the release of some hemicellulose and lignin; (2) the peak that appeared at 1735 cm^−1^ (C=O), indicating hemicellulose degradation; and (3) the peak at 1600 cm^−1^ (C=C aromatic) which showed a decrease in intensity, indicating partial dissolution of lignin. Overall, based on the FTIR spectrum, it can be seen that the treatment of Borassus fruit fiber using 10% NaOH gave the best results, which can clean the content of noncellulose components and maintain the fiber structure. While the 15% NaOH treatment may be too aggressive, risking damaging the main structure of cellulose. These results are in line with the results of the single fiber tensile test, where the results also show that the best tensile strength was obtained when treated using 10% NaOH.

### 3.3. Tensile Properties

The tensile properties of the fibers are presented in Table 2.

The results show that maximum stress, Young's modulus, and % elongation at break increased with NaOH treatment. As mentioned in the literature [16, 23, 24], when the nonpolar material is removed, the diameter of fibers decreases with increased alkali treatment percentages due to the removal of impurities from the fiber surface. Microfibrils have become a more compact fiber bundle due to the formation of new bonds between the cellulose fibril chains, thus improving the tensile properties.

## 4. Conclusion

This work treated Borassus fruit fibers with 5%, 10%, and 15% NaOH solution. The performance of raw and treated Borassus fruit fibers was studied with the perspective of contact angle, SEM observation, FTIR analysis, and tensile properties. As the percentage of NaOH treatment increased, the surface roughness of the fibers increased, as proven by SEM observation analysis. Thus, it affects the contact angle due to removing nonpolar materials such as lignin. The tensile properties of the NaOH-treated fibers increased due to the rearrangement of the fiber structure. Moreover, NaOH treatment caused the reduction in fiber diameter due to the elimination of hydrophobic compounds on the fiber surface, as confirmed by FTIR analysis.

## Figures and Tables

**Table 1:** Diameter of Borassus fruit fiber.

Type	Diameter (μm)		
	Maximum	Minimum	Average
Raw	315.10	285.90	300.82
5% NaOH-treated	289.70	254.35	273.90
10% NaOH-treated	263.20	229.75	243.54
15% NaOH-treated	212.28	200.17	206.88

**Table 2:** Tensile properties of Borassus fruit fibers.

Parameter	Borassus fruit fiber			
	Raw fiber	NaOH-treated		
		5%	10%	15%
Maximum stress (MPa)	430	490	635	560
Young's modulus (MPa)	14.3	15.7	18.8	17
% elongation at break	3.32	3.03	3.33	3.27

## Data Availability

All the observation results were analyzed at Integrated Research and Testing Laboratory, Gadjah Mada University (LPPT UGM).
